# The N‐terminal and C‐terminal halves of histone H2A.Z independently function in nucleosome positioning and stability

**DOI:** 10.1111/gtc.12791

**Published:** 2020-07-22

**Authors:** Shoko Sato, Naoki Tanaka, Yasuhiro Arimura, Tomoya Kujirai, Hitoshi Kurumizaka

**Affiliations:** ^1^ Laboratory of Chromatin Structure and Function Institute for Quantitative Biosciences The University of Tokyo Tokyo Japan; ^2^ Laboratory of Structural Biology Graduate School of Advanced Science and Engineering Waseda University Tokyo Japan; ^3^ Laboratory of Chromosome and Cell Biology The Rockefeller University New York NY USA

**Keywords:** epigenetics, H2A.Z, histone variant, nucleosome positioning, nucleosome stability

## Abstract

Nucleosome positioning and stability affect gene regulation in eukaryotic chromatin. Histone H2A.Z is an evolutionally conserved histone variant that forms mobile and unstable nucleosomes in vivo and in vitro. In the present study, we reconstituted nucleosomes containing human H2A.Z.1 mutants, in which the N‐terminal or C‐terminal half of H2A.Z.1 was replaced by the corresponding canonical H2A region. We found that the N‐terminal portion of H2A.Z.1 is involved in flexible nucleosome positioning, whereas the C‐terminal portion leads to weak H2A.Z.1‐H2B association in the nucleosome. These results indicate that the N‐terminal and C‐terminal portions are independently responsible for the H2A.Z.1 nucleosome characteristics.

## Graphical abstract

The N‐terminal and C‐terminal portions are independently responsible for the H2A.Z.1 nucleosome characteristics. The N‐terminal portion of H2A.Z.1 is involved in multiple positioning of the nucleosome. The C‐terminal portion of H2A.Z.1 leads to weak H2A.Z.1‐H2B association in the nucleosome.

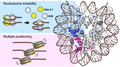

## 1 INTRODUCTION

In eukaryotes, genomic DNA is compactly accommodated within a nucleus. To accomplish this, the genomic DNA binds to basic proteins, termed histones, and forms chromatin (Wolffe, 1998). The nucleosome is the basic unit of chromatin and is composed of two molecules each of histones H2A, H2B, H3 and H4 and approximately 150 base pairs of DNA (Luger, Mäder, Richmond, Sargent, & Richmond, 1997).

There are two categories of histones, canonical and variant (Marzluff, Wagner, & Duronio, 2008; Talbert & Henikoff, 2010, 2017). The canonical histones are produced during the S‐phase of the cell cycle, coupled with DNA replication, and form nucleosomes just after chromatin replication (Marzluff et al., 2008). In higher eukaryotes, the canonical histones are encoded by multiple genes (Marzluff et al., 2008). Histone variants are usually encoded by one or more genes as nonallelic isoforms, and their amino acid sequences are different from those of the canonical histones (Talbert & Henikoff, 2010, 2017). The production of the histone variants is independent of the cell cycle (Talbert & Henikoff, 2017). Histone variants are considered to play specific roles in the regulation of genomic processes, such as DNA replication, repair, recombination and transcription (Giaimo, Ferrante, Herchenröther, Hake, & Borggrefe, 2019; Talbert & Henikoff, 2017).

Each histone variant confers characteristic features on the nucleosome (Koyama & Kurumizaka, 2018; Kurumizaka, Horikoshi, Tachiwana, & Kagawa, 2013). For example, the human testis‐specific histone H3 variants, H3T and H3.5, form nucleosomes with reduced stability, as compared to nucleosomes with the canonical H3 (Tachiwana et al., 2010; Urahama et al., 2016). CENP‐A, an H3 variant accumulating at centromeres, also forms an unstable nucleosome, and the DNA segments at the entry/exit sites of the CENP‐A nucleosome are flexible, as compared with those in the canonical nucleosome (Dechassa et al., 2011; Panchenko et al., 2011; Tachiwana et al., 2011). Other histone variants, such as human H3.Y, mouse H3t and human H2A.B, also form nucleosomes with flexible DNA ends (Arimura et al., 2013; Bao et al., 2004; Doyen et al., 2006; Kujirai et al., 2016; Ueda et al., 2017).

H2A.Z is an evolutionarily conserved histone H2A variant that functions in various biological processes, such as early development and stem cell differentiation (Buschbeck & Hake, 2017; Creyghton et al., 2008; Faast et al., 2001; Giaimo et al., 2019; Hu et al., 2013; Murphy, Wu, James, Wike, & Cairns, 2018; Rangasamy, Berven, Ridgway, & Tremethick, 2003; Talbert & Henikoff, 2010; van Daal, White, Gorovsky, & Elgin, 1988). Remarkably, H2A.Z frequently accumulates around transcription start sites and enhancer elements, indicating its involvement in transcriptional regulation (Albert et al., 2007; Barski et al., 2007; Giaimo et al., 2019; Guillemette et al., 2005; Jin & Felsenfeld, 2007; Li et al., 2005; Raisner et al., 2005; Schones et al., 2008; Zhang, Roberts, & Cairns, 2005). In addition, H2A.Z preferentially localizes in heterochromatin and at DNA double‐strand break sites (Giaimo et al., 2019; Rangasamy et al., 2003; Rangasamy, Greaves, & Tremethick, 2004; Talbert & Henikoff, 2017; Xu et al., 2012). Biochemical studies revealed that H2A.Z forms nucleosomes with multiple positions (Chen et al., 2019; Rudnizky et al., 2016). There are two vertebrate H2A.Z isoforms, H2A.Z.1 and H2A.Z.2 (Coon et al., 2005; Eirín‐López, González‐Romero, Dryhurst, Ishibashi, & Ausió, 2009). H2A.Z.1 forms a nucleosome with lower thermal stability in vitro and higher mobility in living cells, as compared to the canonical nucleosome containing H2A (Horikoshi, Arimura, Taguchi, & Kurumizaka, 2016; Horikoshi et al., 2013).

In the present study, we reconstituted nucleosomes containing human H2A.Z.1 or its swapping mutants, in which the N‐terminal or C‐terminal half of H2A.Z.1 was replaced by the corresponding canonical H2A sequence. We found that the N‐terminal and C‐terminal portions of H2A.Z.1 are independently responsible for the multiple positioning and unstable features of the H2A.Z.1 nucleosome, respectively.

## 2 RESULTS AND DISCUSSION

### 2.1 H2A.Z.1 forms nucleosomes at multiple positions

**FIGURE 1:**
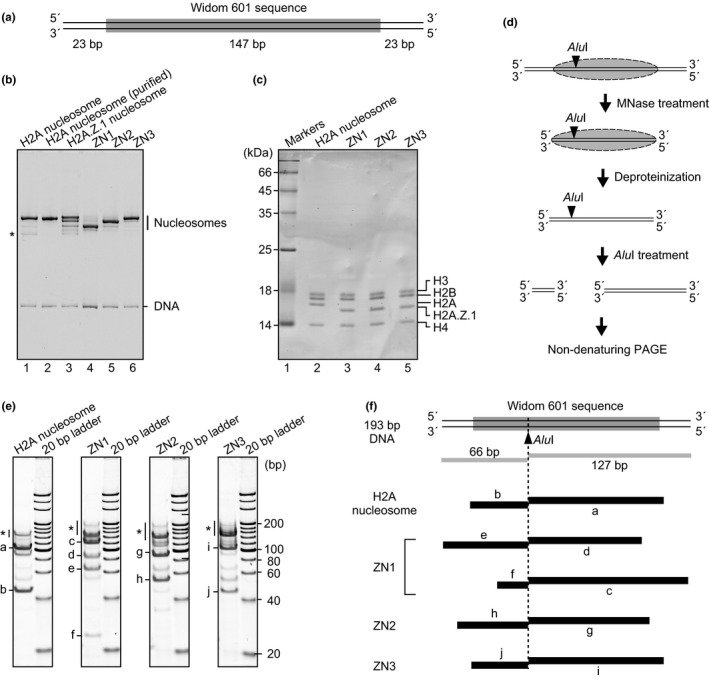
Positioning of the H2A.Z.1 nucleosomes. (a) Schematic representation of the 193 base‐pair DNA containing a 147 base‐pair Widom 601 sequence with 23 base‐pair linker DNA segments. (b) The nucleosomes were analyzed by nondenaturing PAGE with ethidium bromide staining. The band with * indicates a hexasome. We repeated the experiments and confirmed the reproducibility of the results. (c) Histone compositions of separately purified nucleosomes were analyzed by SDS‐PAGE with SYPRO Orange staining. (d) Schematic representation of the *Alu*I*Alu*I restriction enzyme. The DNA fragments were then analyzed by nondenaturing PAGE with SYBR Gold staining. (e) The gel images of the *Alu*I*Alu*I treatment. The bands marked with a‐j correspond to the DNA fragments represented in (F). Estimated lengths (base pairs) of DNA fragments a, b, c, d, e, f, g, h, i and j are about 105, 45, 125, 90, 65, 25, 95, 55, 105 and 45, respectively. (f) Schematic representation of the nucleosome positions for the canonical H2A, ZN1, ZN2 and ZN3 nucleosomes. The lengths of the DNA fragments (a–j) were estimated from the migration distances of the nondenaturing PAGE analysis (e), and the nucleosome positions were mapped on the 193 base‐pair DNA

To study the biochemical characteristics of the nucleosome containing H2A.Z.1, we reconstituted nucleosomes containing human histone H2A.Z.1 or canonical H2A with a 193 base‐pair DNA fragment. This DNA fragment contained a 147 base‐pair Widom 601 sequence with 23 base‐pair linker DNA segments at both termini (Figure 1a and Figure S1). A native polyacrylamide gel electrophoresis (PAGE) analysis demonstrated that the nucleosome containing H2A.Z.1 (H2A.Z.1 nucleosome) migrated as multiple bands (Figure 1b, lane 3). In contrast, the nucleosome containing the canonical H2A (H2A nucleosome) migrated as a single major band with a trace amount of a hexasome band (Figure 1b, lane 1). The three major bands of the H2A.Z nucleosome are termed ZN1, ZN2 and ZN3. We purified the H2A.Z.1 nucleosomes corresponding to ZN1, ZN2, ZN3 (Figure 1b, lanes 4–6) and the major band of the H2A nucleosome (Figure 1b, lanes 2) and determined the histone composition of each nucleosome. All three H2A.Z.1 nucleosomes (ZN1, ZN2 and ZN3), as well as the H2A nucleosome, contained stoichiometric amounts of each histone (Figure 1c). These results indicated that H2A.Z.1 formed nucleosomes with multiple positions, which is consistent with the single‐molecule probing analyses of the reconstituted H2A.Z nucleosome (Chen et al., 2019; Rudnizky et al., 2016).

To determine the nucleosome positioning on the 193 base‐pair DNA, we performed a restriction enzyme (*Alu*I) cleavage assay coupled with micrococcal nuclease (MNase) treatment. Purified ZN1, ZN2 and ZN3 nucleosomes were treated with MNase, which preferentially digests naked DNA, and the DNA fragments protected by the nucleosome formation were analyzed by *Alu*I cleavage (Figure 1d). In the canonical H2A nucleosome, the nucleosome position was mapped at the center of the 193 base‐pair DNA fragment (Figure 1e, left panel, and f). On the other hand, the three H2A.Z.1 nucleosomes were differently mapped: at the DNA ends (ZN1), at about 10 base pairs away from the DNA end (ZN2) and at the same position as the canonical nucleosome (ZN3) (Figure 1e and f). These results indicated that H2A.Z.1 allows the nucleosome to be located at multiple positions.

### 2.2 The N‐terminal half of H2A.Z.1 is responsible for the multiple nucleosome positioning

**FIGURE 2:**
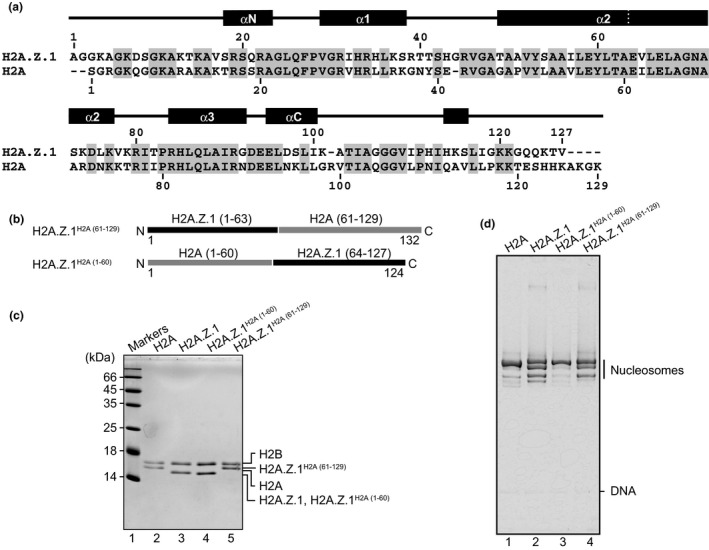
The H2A.Z.1 region responsible for the multiple nucleosome positioning. (a) Sequence alignment of human H2A.Z.1 and canonical H2A. The conserved residues between the two sequences are colored gray. (b) Schematic representation of H2A.Z.1^H2A(61–129)^ and H2A.Z.1^H2A(1–60)^. (c) Purified H2A‐H2B dimers containing canonical H2A, H2A.Z.1, H2A.Z.1^H2A(61–129)^ and H2A.Z.1^H2A(1–60)^ were analyzed by SDS‐PAGE with Coomassie Brilliant Blue staining. (d) The nucleosomes were analyzed by nondenaturing PAGE with ethidium bromide staining. We repeated the experiments and confirmed the reproducibility of the results

In human H2A.Z.1, 41% of the amino acid residues are different from those in the canonical H2A (Figure 2a). To map the H2A.Z.1 region responsible for the multiple nucleosome positioning, we purified the swapping H2A.Z.1 mutants, H2A.Z.1^H2A(61–129)^ and H2A.Z.1^H2A(1–60)^, in which residues 64–127 and 1–63 of H2A.Z.1 were replaced by the corresponding residues 61–129 and 1–60 of canonical H2A, respectively (Figure 2b and c). Therefore, H2A.Z.1^H2A(61–129)^ and H2A.Z.1^H2A(1–60)^ contain the N‐terminal and C‐terminal halves of H2A.Z.1, respectively. Since the amino acid residues of the α2 helices are relatively conserved between H2A.Z.1 and canonical H2A, we placed the swapping site at the center of the α2 helices (Figure 2a). We then reconstituted the nucleosomes with H2A.Z.1^H2A(61–129)^ and H2A.Z.1^H2A(1–60)^ by salt dialysis and determined their nucleosome positioning by a native PAGE analysis. The nucleosome containing H2A.Z.1^H2A(61–129)^ exhibited similar migration profiles to the nucleosome containing wild‐type H2A.Z.1 (Figure 2d, lanes 2 and 4). In contrast, the migration profiles of the nucleosome containing H2A.Z.1^H2A(1–60)^ were similar to those of the canonical H2A nucleosome (Figure 2d, lanes 1 and 3). These results clearly showed that the N‐terminal half of H2A.Z.1 is responsible for the multiple positioning characteristic of the H2A.Z.1 nucleosome.

### 2.3 The C‐terminal half of H2A.Z.1 is responsible for the weak H2A.Z.1‐H2B association with the nucleosome

**FIGURE 3:**
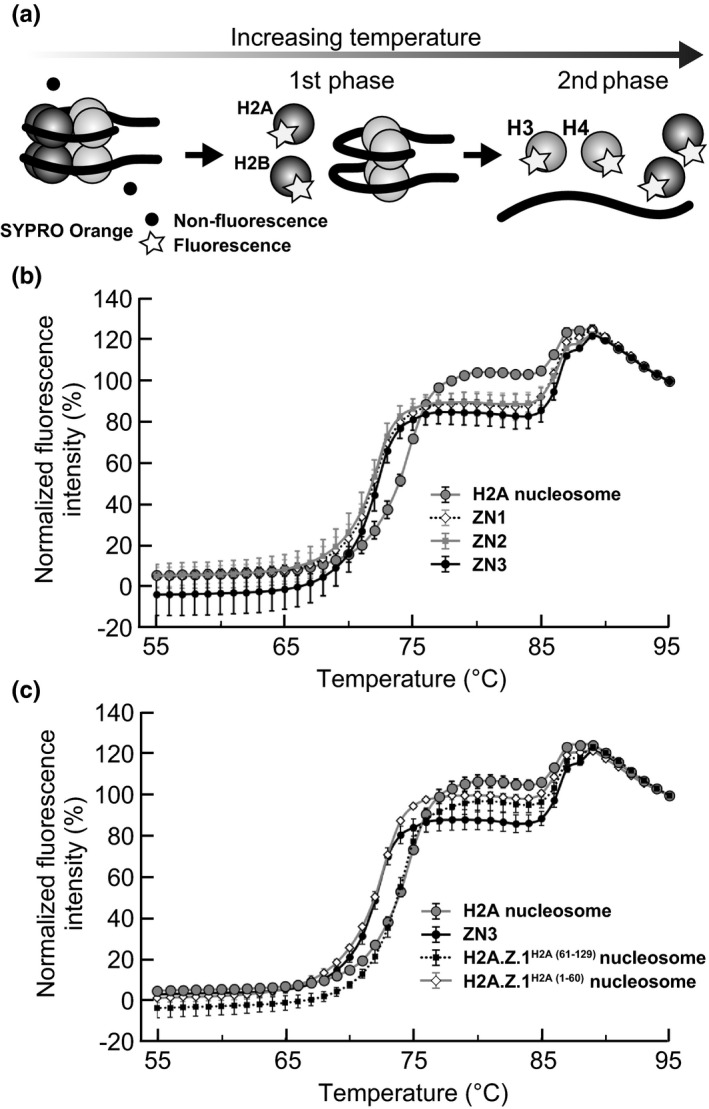
The H2A.Z.1 region responsible for the weak H2A.Z.1‐H2B association with the nucleosome. (a) Schematic representation of the thermal stability assay. The dissociation of the histone proteins from the nucleosome was monitored by the increasing fluorescence intensity. The SYPRO Orange dye emits fluorescence upon binding to the hydrophobic surfaces of thermally denatured histones. (b) Thermal denaturation profiles of the canonical H2A, ZN1, ZN2 and ZN3 nucleosomes. (c) Thermal denaturation profiles of the canonical H2A, ZN3, H2A.Z.1^H2A(61–129)^ and H2A.Z.1 ^H2A(1–60)^ nucleosomes. The averages of the normalized fluorescence intensities of three independent experiments are shown. The error bars indicate standard deviations. Consistent results were obtained by replicated experiments using samples from different nucleosome preparations (Figure S2)

We then tested the stability of the nucleosomes containing H2A.Z.1^H2A(61–129)^ and H2A.Z.1^H2A(1–60)^ by a thermal stability assay (Taguchi, Horikoshi, Arimura, & Kurumizaka, 2014). In this assay, the free H2A‐H2B dissociated from the nucleosome is detected by the binding of the fluorescent probe SYPRO Orange (Figure 3a). The nucleosomes were denatured by a biphasic process. The first phase corresponds to the H2A‐H2B dissociation from the nucleosome, and the second phase represents the H3‐H4 dissociation. The H2A.Z.1‐H2B dimer is dissociated from the nucleosome at a lower temperature than the canonical H2A‐H2B dimer (Horikoshi et al., 2016). Consistently, in all three H2A.Z nucleosomes with different positions (ZN1, ZN2 and ZN3), the first phase was shifted toward a lower temperature, as compared to the H2A nucleosome (Figure 3b). Interestingly, the H2A.Z.1^H2A(61–129)^ nucleosome did not show this lower temperature shift of the first phase and exhibited similar stability to that of the canonical H2A nucleosome (Figure 3c). In contrast, the H2A.Z.1^H2A(1–60)^ nucleosome was clearly more unstable than the canonical H2A nucleosome (Figure 3c). These results indicated that the C‐terminal half of H2A.Z.1 is responsible for the weak H2A.Z.1‐H2B association with the nucleosome.

### 2.4 Possible mechanism for the multiple positioning and instability of the H2A.Z.1 nucleosome

**FIGURE 4:**
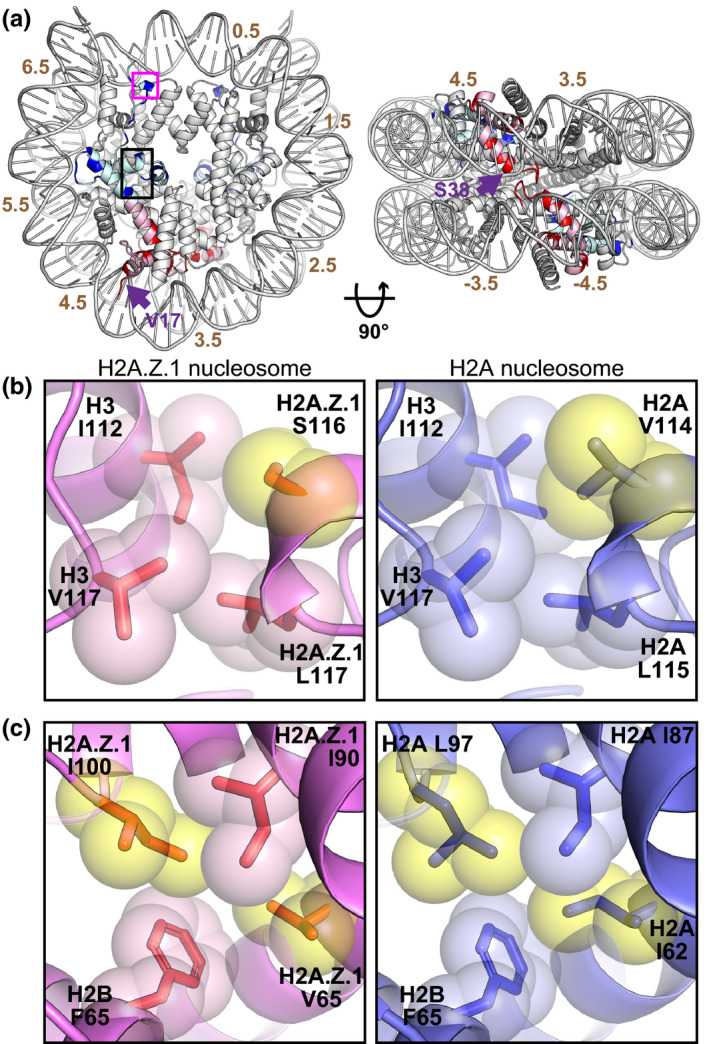
Amino acid residues possibly responsible for the multiple positioning and instability of the H2A.Z nucleosome. (a) Two views of the H2A.Z.1 nucleosome structure (PDB ID: 3WA9). The N‐terminal and C‐terminal halves of H2A.Z.1 are colored pink and light blue, respectively. The residues differing from those of the canonical H2A are colored red in the N‐terminal half and blue in the C‐terminal half of H2A.Z.1. The numbers indicate the superhelical locations of the nucleosomal DNA from the dyad. The H2A.Z.1 Val17 and Ser38 residues are indicated by arrows. The magenta and black boxes represent the hydrophobic clusters shown in close‐up views in (b) and (c), respectively. (b, c) Close‐up views of the regions boxed in (a) (left panels), and the corresponding regions of the canonical H2A nucleosome (PDB ID: 5Y0C, right panels). The atoms of the α‐carbons and side chains of the amino acid residues involved in the hydrophobic clusters are represented as spheres and sticks. The yellow spheres correspond to the different residues between the two molecules

In the present study, we found that the N‐terminal and C‐terminal halves of H2A.Z.1 independently affect the nucleosome positioning and stability, respectively. In the nucleosome, the H2A‐H2B dimers have multiple contact sites with DNA (Koyama & Kurumizaka, 2018). In fact, several H2A.Z‐specific amino acid residues are located near the DNA and on the histone–histone interfaces (Figure 4a, colored blue and red). Among them, the H2A.Z.1 N‐terminal half contacts the DNA backbone around the super helical locations (SHLs) 3.5 and 4.5 of the nucleosome (Figure 4a). In the H2A nucleosome structure, the H2A Lys15 and Lys36 residues are located near the SHL4.5 and SHL3.5 sites, respectively. In H2A.Z.1, these basic H2A Lys15 and Lys36 residues are replaced by the Val17 and Ser38 residues, respectively (Figure 4a). These basic to neutral amino acid substitutions may reduce the histone–DNA interaction and could play roles in inducing the multiple positioning of the H2A.Z.1 nucleosome. It should be noted that the H2A.Z.1 Ser38 residue is substituted by Thr38 in H2A.Z.2 and is responsible for the enhanced mobility of H2A.Z.1 in living cells (Horikoshi et al., 2013). Although the H2A.Z.1 Val17 and Ser38 residues are not highly conserved in the H2A.Z proteins of other species, neutral amino acid residues are conserved at these positions. These neutral amino acid residues may be important for the multiple positioning character of the H2A.Z.1 nucleosome.

Hydrophobic interactions are a major factor in the formation of the stable histone octamer (Taguchi et al., 2014). In the C‐terminal half of H2A, the Val114 and Leu115 residues form a hydrophobic cluster with the H3 Ile112 and Val117 residues (Figure 4b, right panel). In the H2A.Z.1 nucleosome, the H2A Val114 residue is substituted by the hydrophilic Ser116 residue (Figure 4b, left‐right). Other hydrophobic residues, H2A Ile62, Ile87 and Leu97, form a hydrophobic cluster with the H2B Phe65 residue in the H2A nucleosome (Figure 4c, right panel). In the H2A.Z.1 nucleosome, the corresponding residues, Val65, Ile90 and Ile100, also form a hydrophobic cluster with the H2B Phe65 residue, but the interaction may be weaker because the side chain of H2A.Z.1 Val65 residue is shorter than that of the corresponding H2A Ile62 residue (Figure 4c, left panel). These H2A.Z.1‐specific residues located in the C‐terminal half may be important for the weakened H2A.Z.1‐H2B association in the nucleosome. Interestingly, the H2A.Z.1 Val65 and Ile100 residues are conserved between yeasts and mammals. These conserved residues in the hydrophobic cluster may play an important structural role and contribute to the dynamics of the H2A.Z nucleosome.

## 3 EXPERIMENTAL PROCEDURES

### 3.1 Preparation of human histones, histone mutants and histone complexes

The pET15b‐based plasmids for producing the H2A.Z.1 mutants, H2A.Z^H2A(61–129)^ and H2A.Z ^H2A(1–60)^, were prepared by the polymerase chain reaction method with the DNAs of the H2A and H2A.Z.1 expression vectors as templates (Kujirai et al., 2018). The human H2A, H2A.Z.1, H3.1, H4 and the H2A.Z.1 mutants were purified as described previously (Kujirai et al., 2018). Briefly, the histone proteins were produced as His_6_‐tagged proteins in *Escherichia coli* and were purified by affinity chromatography. After removing the His_6_‐tag peptide by thrombin protease treatment, the histone proteins were purified by Mono‐S cation‐exchange chromatography. The proteins were dialyzed against 2 mM 2‐mercaptoethanol and lyophilized. The H2A‐H2B dimer, H2A.Z.1‐H2B dimer, H2A.Z^H2A(61–129)^‐H2B dimer, H2A.Z^H2A(1–60)^‐H2B dimer and H3.1‐H4 tetramer were reconstituted and purified, as described previously (Fujita et al., 2020). Lyophilized H2A and H2B, or H3.1 and H4, were mixed at a 1:1 molar ratio in denaturing solution, containing 7 M guanidine hydrochloride, 20 mM Tris‐HCl (pH 7.5) and 20 mM 2‐mercaptoethanol. The H2A‐H2B dimer and H3.1‐H4 tetramer were reconstituted by refolding, in a solution containing 10 mM Tris‐HCl (pH 7.5), 2 M NaCl, 1 mM EDTA and 5 mM 2‐mercaptoethanol. The histone complexes were purified by Superdex 200 size‐exclusion chromatography (GE Healthcare).

### 3.2 Preparation of histone octamers and nucleosomes

For nucleosome reconstitution, the 193 base‐pair DNA, H2A‐H2B and H3.1‐H4 were mixed at a 1:3.5:1.5 molar ratio, in a solution containing 10 mM Tris‐HCl (pH 7.5), 1 mM dithiothreitol, 1 mM EDTA and 2 M KCl. Nucleosomes were reconstituted by the salt‐dialysis method in 10 mM Tris‐HCl buffer (pH 7.5), containing 1 mM dithiothreitol, 1 mM EDTA and 0.25 M KCl (Dyer et al., 2004; Kujirai et al., 2018). The reconstituted nucleosomes were heated at 55°C for 2 hr and then subjected to a native PAGE analysis, as described below. The reconstituted nucleosomes were separately purified by nondenaturing polyacrylamide gel electrophoresis using a Prep Cell apparatus (Bio‐Rad), in 20 mM Tris‐HCl (pH 7.5) and 1 mM dithiothreitol (Dyer et al., 2004; Kujirai et al., 2018). The separately purified nucleosomes were analyzed by SDS‐PAGE and native PAGE analyses, as described below.

### 3.3 Native PAGE analysis

Nucleosomes (100 ng of DNA in Figure 1b; 1.3 µg of DNA in Figure 2d) were analyzed by nondenaturing 6% polyacrylamide gel electrophoresis in 0.2x TBE (17.8 mM Tris, 0.4 mM EDTA and 17.8 mM boric acid) with ethidium bromide staining. The gel images were obtained using an LAS4000 image analyzer (GE Healthcare).

### 3.4 *Alu*I restriction enzyme digestion assay coupled with MNase treatment

The purified nucleosomes (400 ng of DNA) were treated with MNase (TAKARA, 0.6 units for the H2A nucleosome, 0.7 units for ZN1, 0.5 units for ZN2 and 0.5 units for ZN3) at 25°C for 5 min, in a 20 µL reaction containing 32.5 mM Tris‐HCl (pH 8.0), 10 mM Tris‐HCl (pH 7.5), 25 mM NaCl, 1.25 mM CaCl_2_ and 1.5 mM dithiothreitol. The reactions were stopped by adding 5 µL of stop solution, containing 6.2 mg/ml Proteinase K (Roche), 3.3% SDS and 100 mM EDTA. The DNA fragments were extracted with a Miniprep DNA Purification Kit (Promega). After digestion with 10 units of the *Alu*I (New England Biolabs) restriction enzyme, the DNA fragments were fractionated by nondenaturing 10% polyacrylamide gel electrophoresis, stained with SYBR Gold and detected with an Amersham Typhoon scanner (GE Healthcare). The migration profiles of the DNA fragments were analyzed with the Image Gauge software (GE Healthcare). We then estimated the lengths of the DNA fragments produced by *Alu*I cleavage with the 20 base‐pair DNA markers as references, using the Multi Gauge software (Fujifilm).

### 3.5 Thermal stability assay

The thermal stability of the nucleosomes containing H2A, H2A.Z, H2A.Z^H2A(61–129)^ and H2A.Z ^H2A(1–60)^ was tested, as described previously (Taguchi et al., 2014). Briefly, the purified nucleosome (12 pmol) was mixed with 50 × SYPRO Orange dye (Sigma‐Aldrich) and incubated in a solution containing 20 mM Tris‐HCl buffer (pH 7.5) and 1 mM dithiothreitol. The SYPRO Orange fluorescence emitted by its binding to denatured histones was detected with a StepOnePlus^TM^ Real‐Time PCR system (Applied Biosystems). The temperature gradient ranged from 25°C to 95°C, in steps of 1°C/min. The fluorescence intensity was normalized relative to the fluorescence signal at 95°C.

## Supporting information

Fig S1‐S2
