# Supplementary material for: The N‐terminal and C‐terminal halves of histone H2A.Z independently function in nucleosome positioning and stability
Source: Genes Cells. 2020 Jul 22;25(8):538–46. doi: 10.1111/gtc.12791 (PMC7496805; doi:10.1111/gtc.12791)
Supplement: Supplementary file 1 — Fig S1‐S2 [file GTC-25-538-s001.pdf]

```
1                  20                  40                  60
ATCGGACCCTATCGCGAGCCAGGCCTGAGAATCCGGTGCCGAGGCCGCTCAATTGGTCGT

61  AluI           80                  100                 120
AGACAGCTCTAGCACCGCTTAAACGCACGTACGCGCTGTCCCCCGCGTTTTAACCGCCAA

121                140                 160                 180
GGGGATTACTCCCTAGTCTCCAGGCACGTGTCAGATATATACATCCAGGCCTTGTGTCGC

181         193
GAAATTCATAGAT
```

Supplementary Figure S1. The sequence of the DNA fragment for the nucleosome reconstitution.

The DNA sequence of the 193 base-pair DNA fragment used in the nucleosome reconstitution is presented. The Widom 601 sequence is colored gray, and 23 base-pair linker DNA segments are at both termini.

Supplementary Figure S2

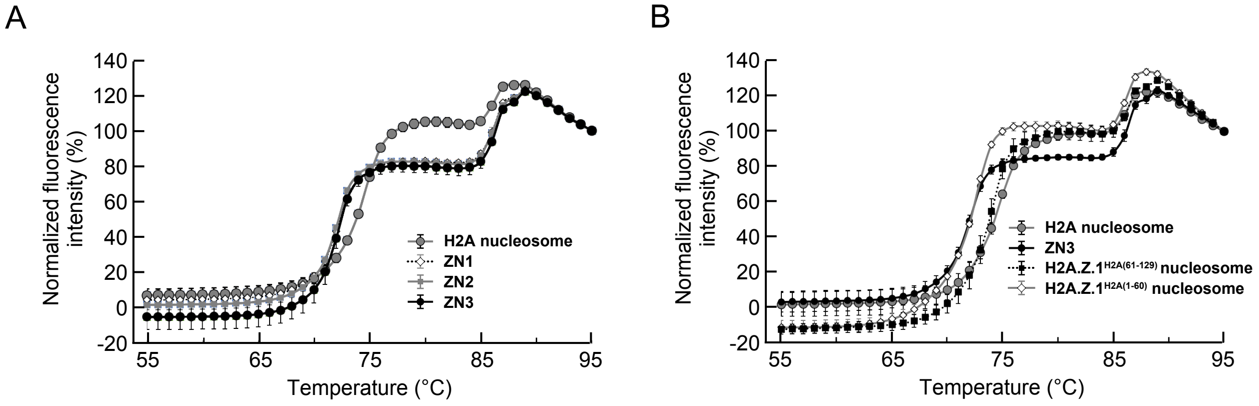

Supplementary Figure S2. Replicated experiments of the thermal stability assay in Figure 3.

(A) Thermal denaturation profiles of the canonical H2A, ZN1, ZN2, and ZN3 nucleosomes. (B) Thermal denaturation profiles of the canonical H2A, ZN3, H2A.Z.1H2A$^{(61-129)}$, and H2A.Z.1 H2A$^{(1-60)}$ nucleosomes. The averages of the normalized fluorescence intensities of three independent experiments are shown. The error bars indicate standard deviations.
